# Machine learning approaches to predict and detect early-onset of digital dermatitis in dairy cows using sensor data

**DOI:** 10.3389/fvets.2023.1295430

**Published:** 2023-11-30

**Authors:** Jennifer Magana, Dinu Gavojdian, Yakir Menahem, Teddy Lazebnik, Anna Zamansky, Amber Adams-Progar

**Affiliations:** ^1^Department of Animal Sciences, Washington State University, Pullman, WA, United States; ^2^Cattle Production Systems Laboratory, Research and Development Institute for Bovine, Balotesti, Romania; ^3^Department of Computer Science, Holon Institute of Technology, Holon, Israel; ^4^Department of Mathematics, Ariel University, Ariel, Israel; ^5^Department of Cancer Biology, University College London, London, United Kingdom; ^6^Tech4Animals Laboratory, Information Systems Department, University of Haifa, Haifa, Israel

**Keywords:** animal behavior, dairy cattle, digital dermatitis, sensor data, machine learning

## Abstract

The present study aimed to employ machine learning algorithms based on sensor behavior data for (1) early-onset detection of digital dermatitis (DD) and (2) DD prediction in dairy cows. Our machine learning model, which was based on the Tree-Based Pipeline Optimization Tool (TPOT) automatic machine learning method, for DD detection on day 0 of the appearance of the clinical signs has reached an accuracy of 79% on the test set, while the model for the prediction of DD 2 days prior to the appearance of the first clinical signs, which was a combination of K-means and TPOT, has reached an accuracy of 64%. The proposed machine learning models have the potential to help achieve a real-time automated tool for monitoring and diagnosing DD in lactating dairy cows based on sensor data in conventional dairy barn environments. Our results suggest that alterations in behavioral patterns can be used as inputs in an early warning system for herd management in order to detect variances in the health and wellbeing of individual cows.

## 1 Introduction

Digital dermatitis (DD) is one of the most prevalent infectious diseases in dairy cows worldwide, being responsible for substantial economic losses due to impaired production and reproduction, higher risks of culling, and treatment costs while having detrimental effects on animal welfare (1–3).

DD in cattle is regarded as a complex disease influenced by multiple microbes. While its exact pathogenesis is still not fully understood (4, 5), foot lesions are often associated with various phylotypes of Treponemes. Consequently, the *Treponema* genus is considered the primary causal agent (6). Other bacteria species such as *Porphyromonas, Fusobacterium*, and *Dichelobacter* are believed to act synergistically (7–9). DD in cattle manifests in the form of ulcerative or growth-like skin lesions, primarily located digitally and on the coronary band of the hoof. The hind legs are affected in over 90% of cases (10), and this condition is typically associated with lameness. It can also coexist with other issues such as foot rot, sole ulcers, sole hemorrhages, and white line disease.

The highly contagious nature and reduced treatment responses of DD (11–13) were shown to result in increased prevalence of up to 91% at the herd level while affecting up to 41% of the animals (14–16). Diagnosis for DD is based on the visual inspection of the feet using the Mortellaro-stage (M-stage) scoring system described and modified by Berry et al. (17), where lesion type and size are differentiated.

Although the etiopathogenesis of bovine DD is not well understood (18), in recent years, several studies have been conducted to identify risk factors associated with the occurrence of DD in dairy cattle. At the individual animal level, the main risk factors for developing DD were found to be breed, milk yield, parity, lactation stage, presence of metabolic diseases, interindividual differences in the immune response, and animal behavior (3, 19, 20), while the main risk factors at the farm level are represented by the housing system, flooring type, plan of nutrition, general farm biosecurity, and preventive practices used to mitigate bovine digital dermatitis (21, 22).

With the recent advent of precision livestock farming (PLF), an increasing body of research addresses machine learning approaches for the early detection of cattle diseases (23–27). Furthermore, several studies have already successfully applied computer vision for detecting and classifying DD in cattle (28–30). As a result, sensor-based behavior monitoring technologies are promising, more affordable, and operationally simpler alternatives for disease monitoring and diagnostics. Currently, a wide range of commercially validated systems are available (31), which monitor behaviors such as feeding, ruminating, activity, and lying. Some of these behavioral patterns have been directly linked to DD in cattle, with ill animals spending more time lying down than their healthy counterparts and devoting less time to feeding and rumination (32, 33). However, to the best of our knowledge, the use of machine learning for the detection of DD from behavioral sensor data has not yet been explored in cattle.

The present study aimed to employ machine learning algorithms based on sensor behavior data for (1) *early-onset detection of DD* and (2) *DD prediction*, with the ultimate goal of setting up early warning tools for DD prediction. These warning tools would then enable farmers and veterinarians to better monitor and manage DD in commercial settings, resulting in a decrease in DD prevalence and severity while improving animal welfare.

## 2 Materials and methods

### 2.1 Animal management and data collection

All procedures involving animals used in the current study were approved by the Washington State University Institutional Animal Care and Use Committee (IACUC), with the approval code ASAF#6770. The data collection process occurred over 60 consecutive days at the Washington State University Knott Dairy Center (KDC) in Pullman, Washington, USA (GPS: 46.6937°N, 117.2423°W).

The experimental cattle facility at the KDC experimental farm houses 180 Holstein pedigreed purebred cows, with lactating animals being housed in a free-stall barn with individual cubicles, using composted manure as bedding. Cows are milked twice per day using a 6×6 ‘herring-bone’ milking parlor, have *ad libitum* access to two water troughs, and are fed a total mixed ration twice per day. The KDC farm practices zero-grazing for lactating cows (indoor housing year-round), with movement alleys and the outside paddock having concrete flooring. During the dry period, the cows are housed in deep-bedded packs with access to grazing areas. Each cow at the KDC experimental farm was fitted with a CowManager® (CowManager B.V., Harmelen, Netherlands) ear tag that continuously records animal behavior, rumination, and ear temperature 24 h per day. The measurements of interest in this study were activity (non-active, active, and highly active), eating time, rumination time, and ear temperature. All behavioral data were calculated as the proportion of time each cow spent exhibiting each behavioral pattern and computed in hours devoted to that behavior per 24 h.

The CowManager® sensor is a molded microchip that has been adapted into a cattle ear identification tag (Supertag; Dalton ID Ltd., Oxfordshire, UK). A three-dimensional accelerometer within the sensor continuously registers the activities of the cow, with the raw data being sent through a wireless connection via routers to a central computer. The raw sensor data are continuously collected, and each minute is classified into one of the four measurement categories: “ruminating,” “eating,” “resting,” and/or “active,” with a proprietary model of the sensor. Data obtained are subsequently expressed as minutes of behavior per hour as well as hours per day and were retrieved through a web-based application. The ear temperature was presented as the average/day and expressed in °C. The sensor used has been previously validated to effectively monitor the behavior of free-stall housed dairy cattle (34).

Cattle were enrolled in the study if they met two criteria: (1) no lesions for at least 7 days prior to the first observation of an active lesion and (2) had at least 2 consecutive days of DD lesion observed. During the study, 21 cows that were between the 1st and 5th lactation periods developed DD. Each cow that developed a DD episode was then matched with a healthy counterpart that had the same parity, reproduction status (open/pregnant), and lactation period (early/mid/late). Lactation periods were classified as early (< 100 days in milk [DIM]), mid (101–199 DIM) or late (> 199 DIM). Therefore, the final dataset included 21 cows with DD and 21 healthy cows. As a prevention method for DD, an acidified copper-, sulfate-, and zinc footbath solution was placed at the exit of the milking parlor. The footbath solution was replaced twice a week, following the recommendations of a hoof specialist. The observer for this study was trained by a hoof specialist to evaluate digital dermatitis (DD) lesions. All hoofs were visually assessed during the first milking in the morning inside the milking parlor, looking exclusively at the hind feet. To date, there has been no golden standard for making observations to determine if a cow has DD. In most on-farm cases, cows are observed for lameness, and those with signs of lameness are further evaluated for hoof disorders. The DD lesion scoring system implemented in this study follows the widely used M-stage scoring system (35). When observed, lesions were categorized as active (red and painful with hair on lesions) or digressing (no hair or little hair, no pain, and scabbing on lesions). Lesion size was categorized as either small (<0.635 cm), medium (0.635–3.81 cm), or large (>3.81 cm) based on the lesion diameter. The same observer recorded the DD status and lesion size daily during the trial to avoid interobserver biases.

### 2.2 Machine learning models

The data used in this study are of time series type, which is a sequence of data points measured at successive points in time spaced at uniform time intervals. All measurements were continuous and aggregated to time frames of a single day. We analyzed 2,520 entries in total (i.e., the daily data about a cow) as part of our dataset. Namely, we analyzed the data, which are the product of 7 days over 6 features, collected for 60 cows. Due to the challenging nature of the problem, we took a two-step approach to tasks of increasing difficulty. The first task was detection, namely whether the cow has DD or not on day 0, looking at data from all days prior to day 0 (−7 days). The second, and a more challenging task, was the prediction/forecasting of DD episodes, especially classifying whether the cow will have DD or not on day 0 based on data x days before day 0 (where the optimal value of x needs to be determined).

### 2.3 Detection machine learning model

The first task we address is providing a machine learning classifier of whether a specific cow has DD or not on day 0. In this section, we describe the training process of the machine learning model. We first divided the dataset into training and testing cohorts such that the training cohort contained 80% of the dataset, while the remaining 20% belonged to the test cohort. Importantly, we ensured the distribution of the target feature in both the training and test cohorts using the Monte Carlo method, taking the best random split out of *n* = 100 attempts. The training cohort was then used to train the model, and the testing cohort was used to evaluate its performance. Importantly, samples from the same individual were either included in the training or testing cohort in order to avoid potential data leakage between the two. Moreover, to make sure the results were robust, we further divided the training cohort using the *k*-fold cross-validation method (36) with *k* = 5. Using the training cohort, we then used the Tree-Based Pipeline Optimization Tool (TPOT) automatic machine learning library method (37). Formally, given a dataset D∈R^r,c^ with c∈N features and r∈N samples, we utilized TPOT, which uses a GA-based approach, to generate and test ML pipelines based on the popular scikit-learn library (38). Formally, we run the TPOT classifier search method to obtain an ML pipeline that aims to optimize the classifier’s mean accuracy over the *k*-folds (39). Once the pipeline was obtained, we further aimed to improve the model’s performance over the training cohort using the grid-search hyperparameters method (40) such that the hyperparameter value ranges were chosen manually (41). Finally, the obtained model was evaluated using the testing cohort. This model development process was similar in nature to other recent studies in sensory data of dairy cattle (42); however, rather than manually testing multiple ML models, we used the automatic machine learning approach, which performed this task more efficiently in terms of time.

### 2.4 Prediction machine learning model

Two important concepts in the context of time series forecasting are ‘lag’ and ‘window’. A ‘lag’ in time series prediction is a way of referencing past data points, e.g., a lag of 1 would mean the previous data point, a lag of 2 would mean the data point two periods back, and so forth. A (rolling) ‘window’ refers to a fixed-size subset of a time series dataset. The aim was to take a portion of the data of a particular length (window size) and move those data across the time series. Having a window allowed us to create aggregated features such as moving averages, sums, and standard deviations. The question, then, becomes, what lag and what window size would yield better performance of the model for prediction? Obviously, with lag 0, we are back to the prediction problem. Going to lags 1, 2, and 3 will decrease our accuracy, but it will also mean that we are able to make the prediction sooner. We thus had a time series task with some lag l∈N and a window size w∈N. In this representation, the disease occurrence prediction takes a binary classification form. However, naturally, the number of negative samples is much larger than the number of possible samples, as these occur once for each cow, by definition. Hence, to balance the data, we undersampled the negatively labeled samples using the K-means method (43) such that the number of clusters equals the number of positive samples. Building on these grounds, we repeat the same computational process as the one used to obtain the disease detection classifier. In addition, to investigate the influence of the lag and window size parameters, the disease occurrence predictor was obtained for all possible combinations of these parameters. To control the balancing method, we also used the class weight fixing method, where the number of samples is kept the same but the weight of each label is different to count for the differences in the labels’ group sizes. Both models were implemented using the Python programming language (version 3.8.1) (44) and set at a value of *p* of ≤0.05 to be statistically significant.

## 3 Results

### 3.1 Digital dermatitis detection on day 0

**Figure 1:**
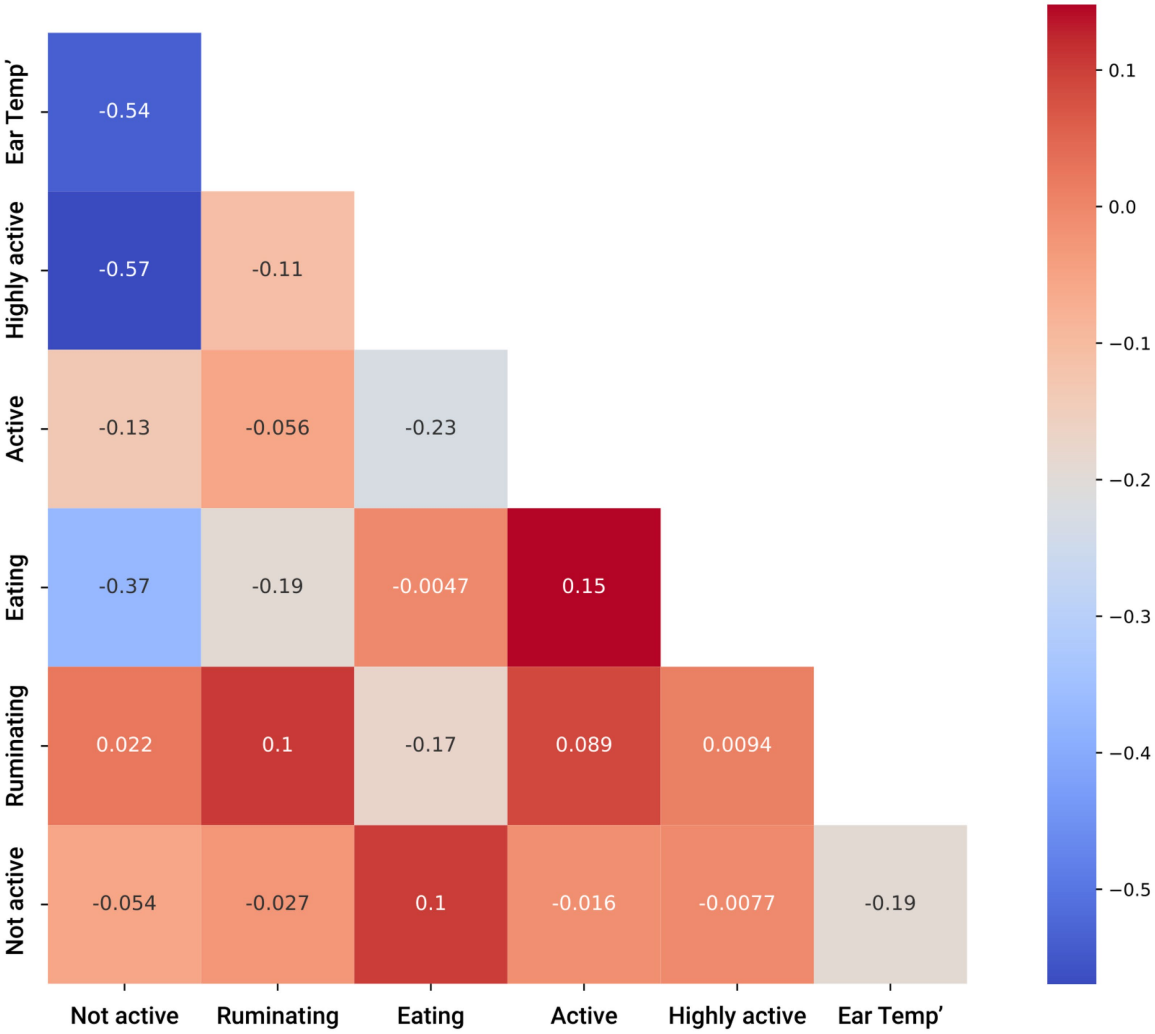
Pearson’s coloration matrix between the input features for the disease detection model.

For a preliminary exploration, we computed the Pearson correlation matrix (45) between the sensor’s data and the target variable (presence/absence of DD in the cow). Figure 1 presents the matrix. As can be noticed, most of the values were less than 0.3, which strongly indicates that the inputted space was mostly linearly independent (46). Hence, a non-linear-based model should be investigated for the proposed challenge.

**Figure 2:**
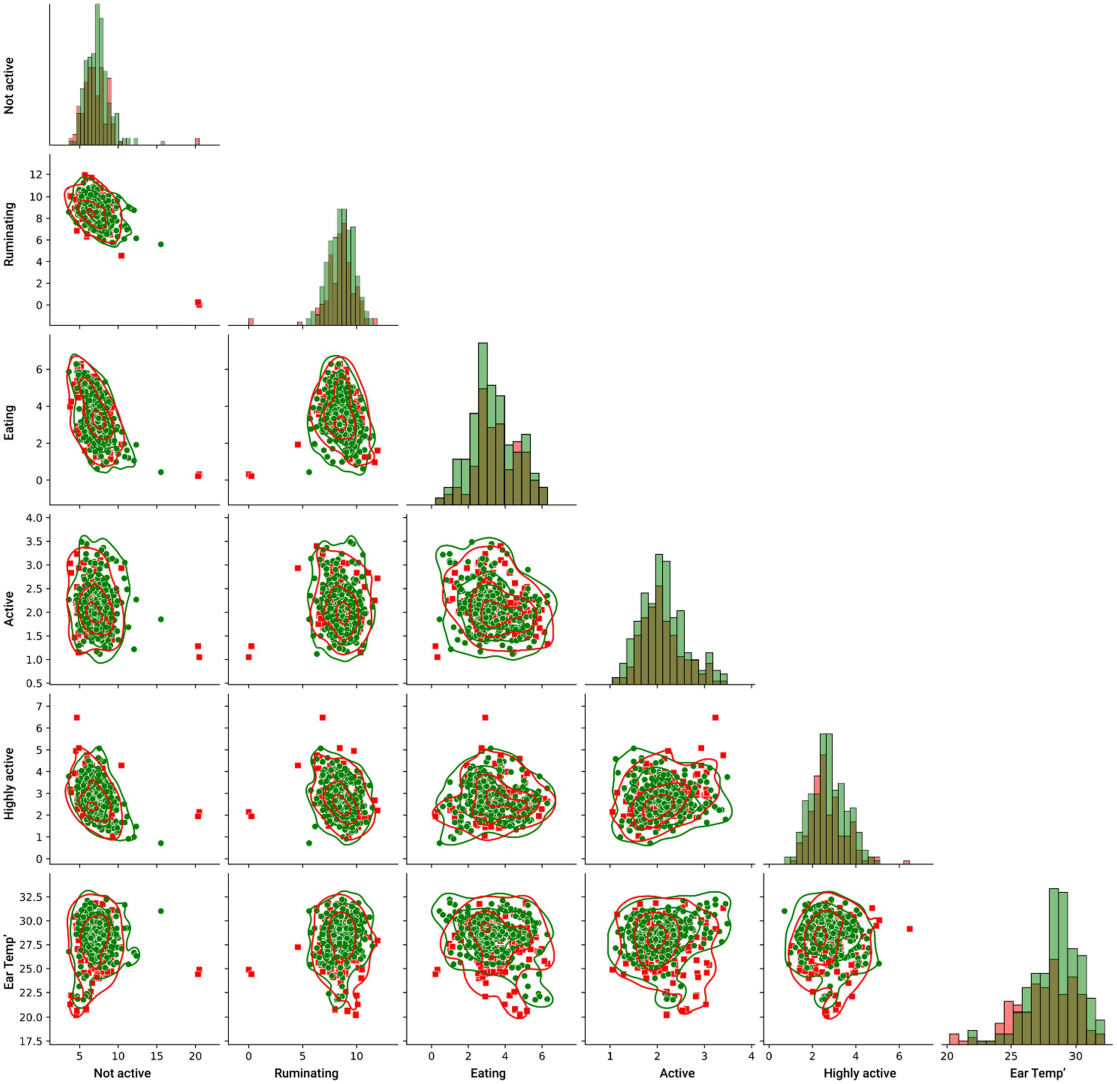
Pair plot between the features of the model, divided by the target features such that the red (square) markers indicate DD sick cows while the green (circle) markers indicate healthy cows. The lines indicate the kernel density estimate of each pairwise distribution.

To this end, we investigated the pairwise relationship of the inputted features and their relationship with the target feature, as presented in Figure 2, such that the red (square) markers indicate DD sick cows while the green (circle) markers indicate healthy cows. The lines indicate the kernel density estimate of each pairwise distribution. In more pairwise plots as well as the features’ histograms, it can be visibly observed that there is no clear separation between the target feature sets.

Based on the above, for disease detection, we obtained an ensemble model that combines a random forest model (47, 48) and a k-nearest neighbors algorithm (49), which received a second-order polynomial extension of the inputted features after min–max normalization (50). For this model, we obtained an accuracy of 81.2% for the training set with 5-fold cross-validation. More importantly, for the testing set, we obtained an accuracy of 79.2%. These results indicate that the proposed model was well-fitting, due to the relatively small difference between the mean performance over the training and testing sets. Nonetheless, the standard deviation of 4.6% indicated that the model might be somewhat non-data stable (51). Considering the standard deviation with a probability of 95% CIs, we estimated that the proposed model would have an accuracy of at least 72%.

**Figure 3:**
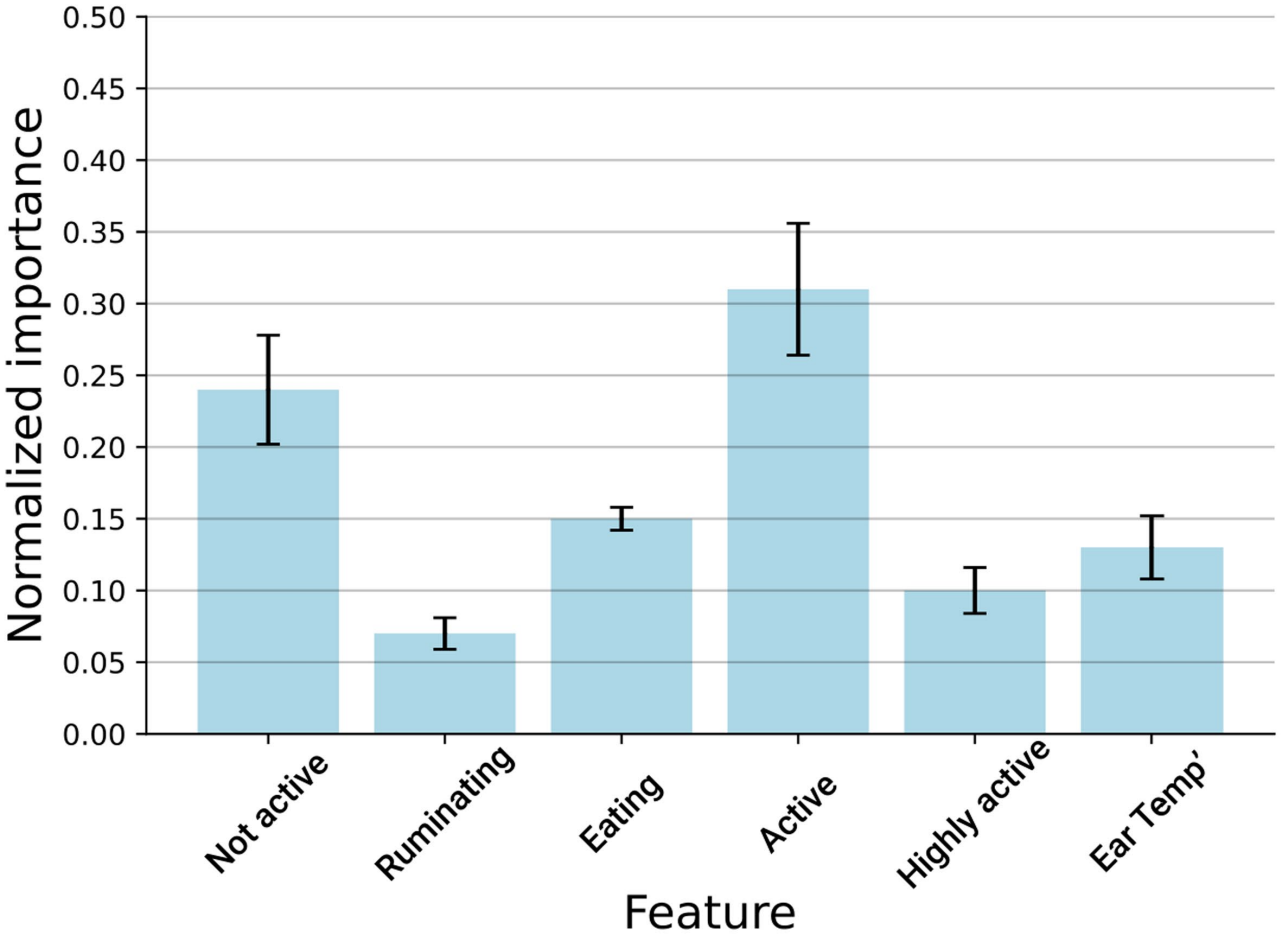
Disease detection model’s feature importance measures the relative information gained from each feature. The results are shown as the mean ± standard deviation of 5-fold cross-validation performed on the entire dataset.

To learn which features contribute the most to the model’s classification capabilities, we computed the model’s features by removing one feature at a time, evaluating how this influences the model’s accuracy, and normalizing these results once all values were obtained. We repeated this process on the entire dataset with a 5-fold cross-validation. Figure 3 shows the results of this analysis, where it can be observed that ‘activity’ is the most important feature, followed by the ‘not-active’ feature.

**Figure 4:**
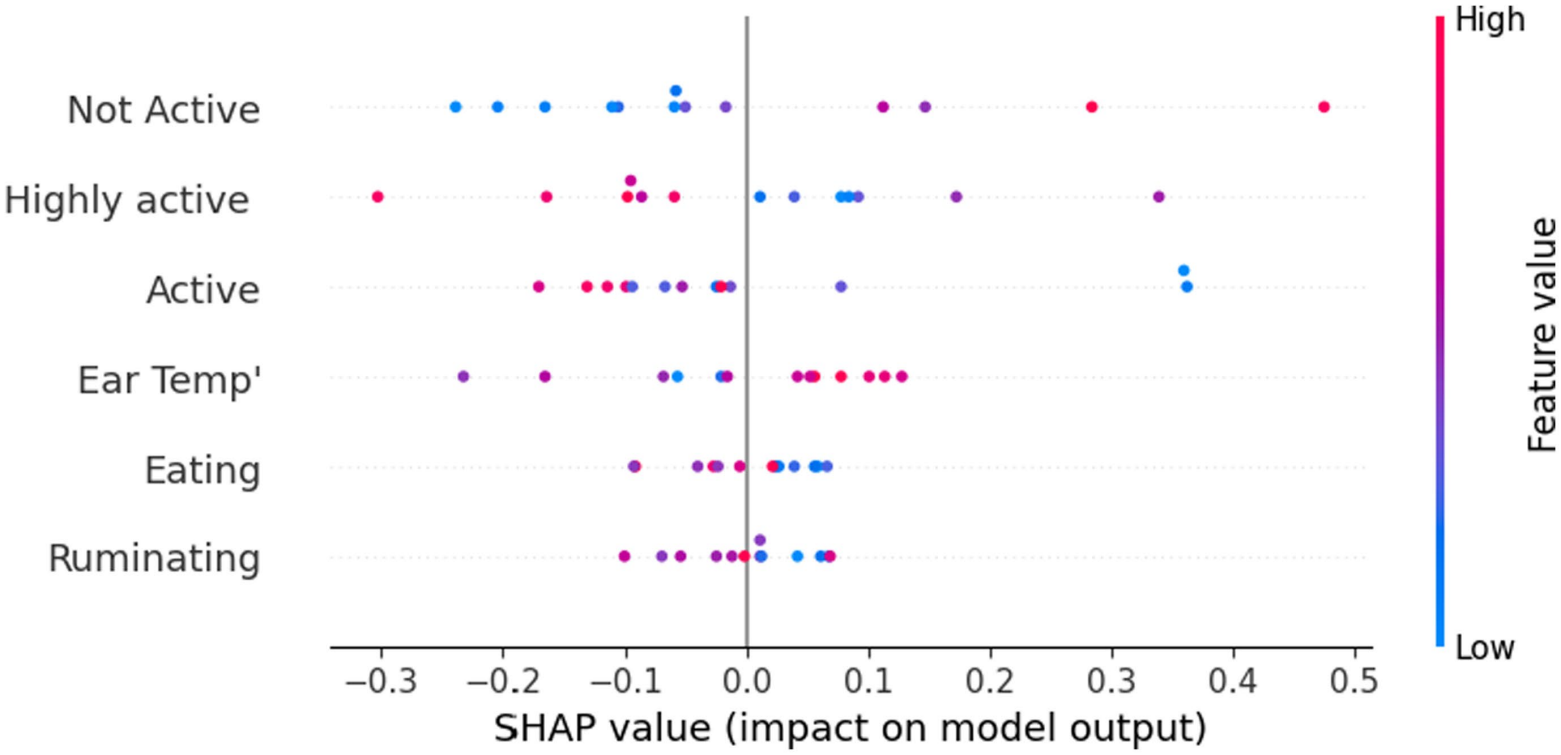
Disease detection model’s feature importance is measured by the SHapley Additive exPlanations (SHAP) value of each feature.

In a complementary manner, Figure 4 presents the feature that is important using the SHapley Additive exPlanations (SHAP) value, which connects optimal credit allocation with local explanations using the classic Shapley values from game theory. Specifically, it shows how the value of each feature corresponds to the model’s prediction. One can notice that higher values of ear temperature and non-activity are associated with a higher probability of DD occurrence.

### 3.2 Digital dermatitis prediction prior to day 0

**Figure 5:**
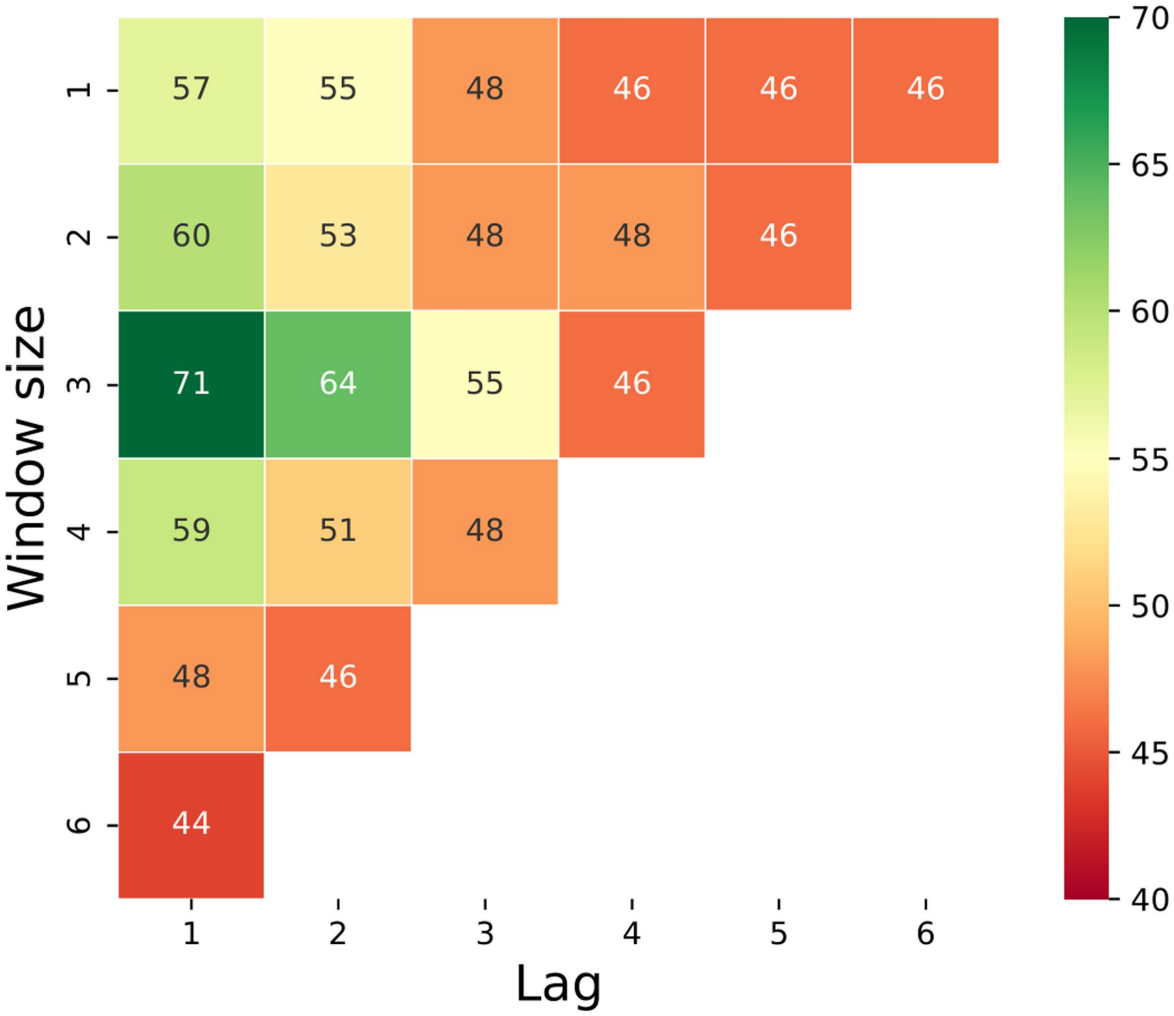
Heatmap of the models’ accuracy on the test set (presented in percentage) as a function of their lag and window size. Notably, a 50% accuracy of a binary prediction indicates a random choice; thus, all results below shows that the model failed to learn any significant pattern.

To find the optimal parameters for window and lag, Figure 5 shows a sensitivity analysis of the model’s accuracy, which was computed for the test set as a function of the lag and window size of the prediction. We can see that, for example, 2 days prior to the appearance of the first clinical signs, we have an accuracy of 64% by looking at a window from 3 days ago. One day prior to day 0, the accuracy increases to 71% (with a window of 3 days). It is important to point out that a 50% accuracy of a binary prediction, such as the one presented in this case, indicates a random choice, thus taking into account that a larger window or looking more days ahead yields low performance, indicating that the model failed to learn any significant pattern and, as a result, more or less guessing the result with some minor (false) bias obtained from slightly overfitting of the training set. In addition, the results are comparable as we downsampled the train and test set sizes to be identical for all cases such that the train and test sets include 98 and 28 samples, respectively.

## 4 Discussion

In this study, we present a machine learning model for DD detection on the first day for the appearance of clinical signs with an accuracy of 79% and a model for the prediction of DD 2 days prior to the appearance of the first clinical signs with an accuracy of 64%. The accuracy attained for the detection of DD was higher in our study when compared to reports by Cernek et al. (28), which applied computer vision approaches for detecting DD in cattle.

In the current study, activity was found to be the most important sensor feature for DD detection. Similarly, Tsai et al. (52) reported that activity and, most importantly, changes in time devoted to walking represent valid indicators for disease detection in cattle. The same authors outline that the current use of PLF needs an improvement in detection accuracy at the farm level. Our results are also in line with the findings by Soriani et al. (53), who reported changes in lying time for cows affected by lameness. Contrary to our findings, these authors found a significant decrease in the time devoted to ruminating during the first days of subclinical diseases or health disorders.

Barker et al. (54) validated the combined use of an animal neck-mounted sensor with a location device to classify cattle behavior, with feeding behavior patterns being used for lameness detection. Interestingly, feeding behavior has not played a significant role in DD detection or prediction in our case. This can be explained by the differences in sensor devices, as well as differences in the machine learning models used, and deserves further exploration.

Regarding temperature variations, Harris-Bridge et al. (55) found a significant temperature rise at the foot level in dairy cows with DD using infrared thermography, with similar results reported by Pirkkalainen et al. (56) for rectal temperature in DD vs. healthy cows, with authors attributing the temperature rises to the effects of inflammation at the foot level. However, such temperature fluctuations have not been observed in our study, most likely due to the sensor being placed in the animal’s ear, and thus, the assumed temperature rise in cows with DD might have occurred only in the plantar region.

The results of our study highlight the potential applications of behavioral sensor data extracted from commercially available sensors for the prediction of digital dermatitis. Current findings are in accordance with those reported by Benaissa et al. (57), which found that cattle sensor behavior data are strongly linked to the animals’ health and welfare. Furthermore, Hosseininoorbin et al. (58) found that both lameness and infectious diseases can be detected via the use of cattle behavior. However, further studies are needed to expand this exploration, focusing on studying the forecasting parameters of lag and window revealed in this study. The use of other, more complex sensor systems that provide more fine-grained behavioral data can potentially increase the performance of the machine learning models presented here.

The main drawback for the lack of adoption of sensor data combined with other PLF systems in commercial settings is the additional cost to be incurred for the farms. It is important to note that farmers are more likely to adopt a PLF system if the system provides useful information they can use to make informed decisions. The activity monitoring device used in the current study is currently used by farmers to identify potentially unhealthy animals; however, the system is not able to differentiate between different types of sickness. One of our intentions for this study is to demonstrate how activity monitoring device data can be used to identify cows with DD. If the system could incorporate this type of alert for cows with DD, the system would have added value and may be more widely adopted. Overcoming bottlenecks such as user adoption could result in better monitoring of the herd by improving estrous and early disease detection, which would then translate into improved overall farm efficiency. For instance, in a study that coupled accelerometer and GPS location data, Cabezas et al. (59) found a high accuracy of 93% for classifying four dairy cattle behavioral patterns. The grouping of these two sensors was also used to track the social interactions between cows and social behavior, which was significantly linked to both health and animal welfare (60). These aspects are of high importance, given that Proudfoot et al. (61) reported their observation of sick cows isolating themselves and avoiding both allogrooming and agonistic interactions with herd-mates, mainly throughout the use of less frequented cubicles located at the far ends of the barn and away from resources such as feeding alleys and water troughs.

This study is not without limitations. First, the number of developed DD cases during the trial-period and that qualified for enrolment in the study-herd could be increased, hopefully leading to higher performance for both detection and prediction models. Therefore, for our future studies, we plan to include more farms with different barn designs and test the machine learning models in more diverse farming settings. Furthermore, the currently commercially available behavior sensors are focused on monitoring a rather limited number of behavioral patterns, providing data mainly on feeding, ruminating, and activity time budgets. The detection of behaviors that are less frequent or are being expressed during shorter periods of time, such as social interactions, the resting position of the animal, or drinking bouts and rates, and even changes in the behavioral circadian rhythm could be altered during a disease episode; however, to date, the validation of sensors to monitor such behaviors remains a challenge. To overcome these shortcomings, several authors recommend the integrated use of additional PLF tools, such as image analysis-based systems, pressure sensors, radio-frequency identification, and ultra-wideband technology (62–64); thus, progress on this front is expected.

## 5 Conclusion

In conclusion, a machine learning model that is capable of predicting and detecting bovine digital dermatitis in cows housed under free-stall conditions based on behavior sensor data has been proposed and tested in this exploratory study. The model for DD detection on day 0 of the appearance of the clinical signs has reached an accuracy of 79%, while the model for the prediction of DD 2 days prior to the appearance of the first clinical signs has reached an accuracy of 64%. The proposed machine learning models might help to achieve a real-time automated tool for monitoring and diagnosing DD in lactating dairy cows based on behavior sensor data in conventional dairy barn environments. Our results suggest that alterations in behavioral patterns at individual levels can be used as inputs in an early warning system for herd management in order to detect variances in the health and wellbeing of individual cows.

## Data availability statement

The raw data supporting the conclusions of this article will be made available by the authors, without undue reservation.

## Ethics statement

All procedures used in the current study were approved by the Washington State University Institutional Animal Care and Use Committee (IACUC), approval code ASAF#6770. The studies were conducted in accordance with the local legislation and institutional requirements. Written informed consent was obtained from the owners for the participation of their animals in this study.

## Author contributions

JM: Formal analysis, Investigation, Writing – original draft. DG: Data curation, Formal analysis, Funding acquisition, Investigation, Resources, Writing – review & editing. YM: Data curation, Formal analysis, Methodology, Validation, Investigation, Writing – original draft. TL: Data curation, Formal analysis, Investigation, Methodology, Software, Writing – original draft. AZ: Data curation, Formal analysis, Investigation, Methodology, Software, Validation, Writing – review & editing. AA-P: Conceptualization, Methodology, Project administration, Resources, Supervision, Validation, Writing – review & editing.
